# Therapeutic Potential of Mesenchymal Stem Cells for Cancer Therapy

**DOI:** 10.3389/fbioe.2020.00043

**Published:** 2020-02-05

**Authors:** Abdelkrim Hmadcha, Alejandro Martin-Montalvo, Benoit R. Gauthier, Bernat Soria, Vivian Capilla-Gonzalez

**Affiliations:** ^1^Andalusian Center for Molecular Biology and Regenerative Medicine (CABIMER), Pablo de Olavide University, University of Seville, CSIC, Seville, Spain; ^2^Biomedical Research Network on Diabetes and Related Metabolic Diseases (CIBERDEM), Institute of Health Carlos III, Madrid, Spain; ^3^School of Medicine, Miguel Hernández University, Alicante, Spain; ^4^Pablo de Olavide University, Seville, Spain

**Keywords:** mesenchymal stem cells, cancer, cell therapy, therapeutic agents, anti-tumor activity

## Abstract

Mesenchymal stem cells (MSCs) are among the most frequently used cell type for regenerative medicine. A large number of studies have shown the beneficial effects of MSC-based therapies to treat different pathologies, including neurological disorders, cardiac ischemia, diabetes, and bone and cartilage diseases. However, the therapeutic potential of MSCs in cancer is still controversial. While some studies indicate that MSCs may contribute to cancer pathogenesis, emerging data reported the suppressive effects of MSCs on cancer cells. Because of this reality, a sustained effort to understand when MSCs promote or suppress tumor development is needed before planning a MSC-based therapy for cancer. Herein, we provide an overview on the therapeutic application of MSCs for regenerative medicine and the processes that orchestrates tissue repair, with a special emphasis placed on cancer, including central nervous system tumors. Furthermore, we will discuss the current evidence regarding the double-edged sword of MSCs in oncological treatment and the latest advances in MSC-based anti-cancer agent delivery systems.

## Introduction

Mesenchymal stem cells (MSCs), also referred to as mesenchymal stromal cells, are adult stem cells capable of self-renewal and multilineage differentiation (Jiang et al., 2002). They were originally found in the bone marrow (Friedenstein et al., 1970), but they were later identified in other tissues including adipose tissue, muscle, peripheral blood, hair follicles, teeth, placenta and umbilical cord (da Silva Meirelles et al., 2006). Although MSCs may exhibit different characteristics depending on their tissue of origin, they must meet the three minimal criteria defined by the International Society for Cellular Therapy (ISCT) (Dominici et al., 2006). First, MSCs must show plastic-adherence when grown *in vitro*. Second, MSCs must express the surface antigens CD73, CD90, and CD105 while lacking expression of CD45, CD34, CD14 or CD11b, CD79α or CD19 and HLA-DR. Third, MSCs must differentiate into mesodermal cell types (i.e., adipocytes, chondrocytes, and osteoblasts) when cultured under specific conditions. In addition to mesodermal linage, MSCs are capable of differentiating into cells of non-mesodermal origin (i.e., ectodermal and endodermal lineages), such as neuronal cells, cardiomyocytes, hepatocytes or epithelial cells (Lee et al., 2004; Paunescu et al., 2007; Quevedo et al., 2009; Gervois et al., 2015). This plasticity of MSCs confers benefits in tissue regeneration.

**FIGURE 1:**
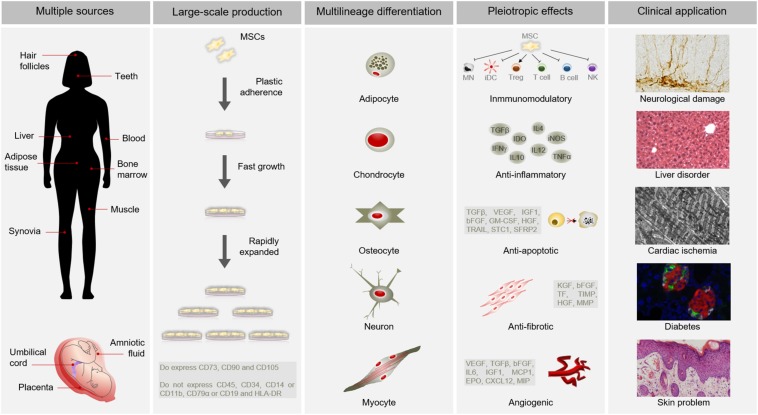
Advantages of MSCs for clinical use. MSCs possess multiple advantages for clinical application. Among other benefits, MSCs can be isolated from several sources, are large-scale produced, differentiate into a variety of cell types and have pleiotropic effects. All these advantages make MSCs suitable for clinical application in different pathological conditions, such as neurological damages, liver disorders, cardiac ischemia, diabetes or skin problems. Abbreviations: HLA-DR, major histocompatibility complex class II DR; MN, monocyte; iDC, immature dendritic Cell; Treg, Regulatory T cell; NK, natural killer cell; TGFβ, transforming growth factor; INFγ, interferon γ; IDO, indoleamine 2,3-dioxygenase; IL10, interleukin 10; IL4, interleukin 4; IL12, interleukin 12; iNOS, inducible nitric oxide synthase; TNFα, tumor necrosis factor α; VEGF, vascular endothelial growth factor; IGF1, insulin like growth factor 1; bFGF, basic fibroblast growth factor; GM-CSF, granulocyte macrophage colony-stimulating factor; HGF, hepatocyte growth factor; TRAIL, TNF-related apoptosis-inducing ligand; STC1, stanniocalcin 1; SFRP2, secreted frizzled related protein 2; KGF, keratinocyte growth factor; TF, tissue factor; TIMP, tissue inhibitor of metalloproteinases; MMP, matrix metalloproteinases; IL6, interleukin 6; MCP1, monocyte chemoatractant protein 1; EPO, erythropoietin; CXCL12, C-X-C motif chemokine 12; MIP, macrophage inflammatory protein.

Mesenchymal stem cells have become as the top used stem cell type for clinical application due to numerous advantages (Connick et al., 2012; Gotherstrom et al., 2014; Karantalis et al., 2014; Rushkevich et al., 2015; Thakkar et al., 2015; Vega et al., 2015; Fernandez et al., 2018) (Figure 1). In addition to different source and multilineage differentiation potential, MSCs also possess the capacity to migrate to injured sites in response to environmental signals and promote tissue regeneration mediated by the release of paracrine factors with pleiotropic effects. Through interaction with the host niche, MSCs are able to inhibit the immune system, promote cell survival, or induce angiogenesis among others pleiotropic activities (Salgado et al., 2010). Of these, the immunosuppressive role of MSCs is particularly interesting for clinical use since it confers resistance to rejection by the host immune system after transplantation. Furthermore, MSCs can be obtained from easily accessible sources by minimally invasive methods (e.g., peripheral blood, adipose tissue) and can be rapidly expanded in large-scale for clinical use (Escacena et al., 2015). This allows to produce a patient-specific medicinal product (i.e., autologous medicinal product) within a therapeutic time window. In addition, the possibility of obtaining MSCs from adult tissue circumvent the ethical issues associated with the use of embryonic source (Lo and Parham, 2009; Ramos-Zuriga et al., 2012). All these advantages of MSCs make this cell type a powerful tool for clinical application in regenerative medicine.

Although MSCs have shown tremendous therapeutic potential in various diseases, their application for cancer treatment remains controversial. While some studies indicate that MSCs may contribute to cancer pathogenesis, emerging data support the beneficial effects of MSCs for oncological treatment. In this review, we provide an overview on the therapeutic application of MSCs for regenerative medicine and discuss the double-edged sword of MSCs for cancer.

## Therapeutic Potential of MSCs

Over the past decades, a large number of studies have emerged using MSC-based therapies in preclinical studies to treat many different pathologies, including neurological disorders, cardiac ischemia, diabetes and bone and cartilage diseases (Si et al., 2012; van Velthoven et al., 2013; Jones et al., 2015; Liu et al., 2016; Ozeki et al., 2016; Capilla-Gonzalez et al., 2018; Chau et al., 2018; Rehorova et al., 2019; Soria et al., 2019). The therapeutic potential of MSCs is firstly mediated by their inherent ability to migrate toward damaged tissues. Then, engrafted cells secrete bioactive mediators, such as growth factors, cytokines and extracellular vesicles that exert immunosuppressive, anti-apoptotic, anti-fibrotic, angiogenic, and anti-inflammatory effects (Salgado et al., 2010). For instance, a study using a neonatal stroke rat model showed that intranasal delivery of bone marrow MSCs reduces infarct size, gray-white matter loss, and motor deficits (van Velthoven et al., 2013). These beneficial effects were in part explained by an increased cell proliferation in the ischemic hemisphere of transplanted rats. In a mouse model of Friedreich’s ataxia, intrathecal injections of bone marrow MSCs improved motor function and delayed neurodegeneration by releasing the neurotrophic factors Neurotrophin-3, Neurotrophin-4, and brain-derived neurotrophic factor, which are implicated in neuronal survival (Jones et al., 2015). Human MSCs derived from umbilical cord showed benefits by improving ventricular function in a porcine model of myocardial ischemia (Liu et al., 2016). In this study, the authors described that MSC-treated pigs exhibit increased angiogenesis, reduced apoptosis and decreased fibrosis in the ischemic heart. Bone marrow-derived MSCs have also shown benefits in improving insulin sensitivity associated with an increased GLUT4 expression in type 2 diabetic rats (Si et al., 2012). More recently, the intranasal application of human adipose-derived MSCs was found to prevent neurocognitive impairments induced by cranial radiation in mice (Soria et al., 2019). The neuroprotective role of intranasally delivered MSCs was mediated by limiting pro-inflammatory processes, restricting oxidative damage accumulation, and reducing neuronal loss after radiation. Another study reported beneficial effects of umbilical cord-derived MSC extracts for atopic dermatitis in a murine model by reducing the T cell response (Song et al., 2019). These reports uncover two main properties of MSCs that determine their therapeutic potential; the capacity to migrate toward the lesion site and the ability to repair damaged tissues.

### Migration Toward Damaged Tissues

**FIGURE 2:**
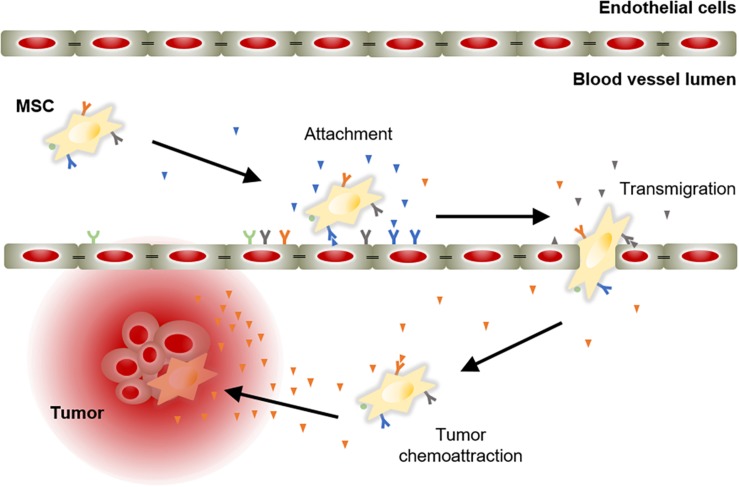
Model of chemoattractant-induced MSC migration toward tumor lesion. Mobilization of MSCs initiates with their incorporation into the circulation. Then, MSCs migrate via the blood stream to areas of injury in response to chemoattractant cues. Ligand-receptor bindings allow MSCs to attach to endothelial cells lining the blood vessels. Subsequently, MSCs activate and initiate the process to cross the endothelium to move toward the target tissue, guided by a chemotactic gradient.

The success of an advanced therapy medicinal product initially depends on its ability to reach target tissues. MSCs possess inherent tropism toward damaged sites that is controlled by a large number of factors and mechanisms, including chemoattractant signals. For instance, the C-X-C motif chemokine ligand 12 (CXCL12) is a frequent triggering factor at the site of injury. It has been demonstrated that a subpopulation of MSCs expresses the C-X-C chemokine receptor type 4 (CXCR4) that binds to its ligand, the CXCL12, to mediate cell migration (Wynn et al., 2004; Ma et al., 2015). Aside from CXCR4, MSCs express other chemokine receptors, such as CCR1, CCR2, CCR4, CCR7, CCR8, CCR9, CCR10, CXCR1, CXCR2, CXCR3, CXCR4, CXCR5, CXCR6, and CX3CR1 (Sordi et al., 2005; Von Luttichau et al., 2005; Honczarenko et al., 2006; Ringe et al., 2007). These receptors are essential to respond to triggering factors at the site of injury. In addition, MSCs also express cell adhesion molecules, including CD49d, CD44, CD54, CD102, and CD106 (De Ugarte et al., 2003). These chemokines and cell adhesion molecules orchestrate the mobilization of MSCs to sites of injury, in a similar manner to white blood cells do (Kolaczkowska and Kubes, 2013). MSC mobilization is a multistep process that encompasses the attachment of free circulating MSCs in the blood stream to transmigrate between endothelial cells with the ultimate goal of migrate and engraft to the target tissue (Figure 2).

### Tissue Repair Ability

Once recruited in the injured site, MSCs contribute to tissue repair and regeneration through activation of several mechanisms. A growing body of research has demonstrated that MSCs display pleiotropic effects, which give them an enormous therapeutic potential (Figure 1). In response to injury signals, MSCs secrete a variety of mediators of tissue repair, including anti-apoptotic, anti-inflammatory, immunomodulatory, anti-fibrotic and angiogenic agents (Caplan and Dennis, 2006; Meirelles Lda et al., 2009; Maltman et al., 2011; Escacena et al., 2015). Among pleiotropic effects, anti-inflammatory and immunomodulatory properties are mainly responsible for the therapeutic benefits of MSCs. As sensors of inflammation, MSCs release soluble factors, such as transforming growth factor β (TGFβ), Indoleamine 2,3-dioxygenase (IDO), Tumor Necrosis Factor α (TNFα), Interleukin 10, and Interferon gamma (INFγ), which interfere with the immune system and modify the inflammatory landscape (Prockop and Oh, 2012). Pivotal studies showed that MSCs inhibit the proliferation of T and B cells (Di Nicola et al., 2002; Corcione et al., 2006; Song et al., 2019), suppress the activation of natural killer cells (Sotiropoulou et al., 2006), and prevent generation and maturation of monocyte-derived dendritic cells (English et al., 2008; Spaggiari et al., 2009). Furthermore, MSCs are able to promote the generation of regulatory T cells (Maccario et al., 2005), which exert immunosuppressive effects. Although soluble factors play a key role in the immunosuppressive activity of MSCs, cell-to-cell contact also influences immune responses (Ren et al., 2010; Li Y. et al., 2019). For instance, direct contact between MSCs and proinflammatory macrophages has been shown to induce immune tolerance through induction of tumor necrosis factor-stimulated gene-6 (TSG-6) production (Li Y. et al., 2019). MSC-mediated modulations of the immune response set in motion essential inflammatory processes that significantly promote tissue repair and regeneration by driving healing, scarring and fibrosis (Julier et al., 2017).

Another typical property of MSCs that is involved in their therapeutic effects is the multilineage differentiation potential. In addition to mesodermal linage, MSCs can differentiate into cells of ectodermal and endodermal origin, such as neuronal cells, cardiomyocytes, hepatocytes or epithelial cells (Lee et al., 2004; Paunescu et al., 2007; Quevedo et al., 2009; Gervois et al., 2015). This ability to differentiate into cell types of non-mesodermal origin has been questioned by researchers claiming that differentiated cells from MSCs are able to dedifferentiate and transdifferentiate into cells of another developmental lineage (Song and Tuan, 2004). Notwithstanding, the versatile differentiation potential of MSCs allows the replacement of damaged or dead cells from different tissues. However, several studies indicate that, after administration, MSCs transiently engraft at the injury site for a short period of time and then disappear. The latter suggest that MSCs must activate mechanisms in the host niche which contribute to tissue repair. For instance, the cross-talk between MSCs and the damaged tissue microenvironment results in the secretion of specific agents involved in proliferation and differentiation of local precursor cells. In this context, a study suggested that systemic administration of MSCs improves radiation-induced intestinal epithelium injury in mice, by increasing the activation of the Wnt/β-catenin signaling pathway that drives proliferation and maintenance of intestinal stem cells (Gong et al., 2016). In a mouse models of Alzheimer’s disease, intravenous administration of MSCs stimulated proliferation and differentiation of hippocampal neuronal progenitor cells into mature neurons by increasing the Wnt signaling pathway (Oh et al., 2015).

## Divergent Roles of MSCs in Cancer Treatment

**FIGURE 3:**
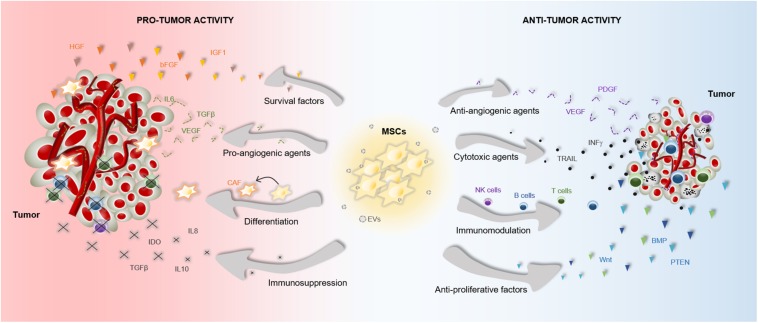
Pro- and anti-tumor effects of MSCs. The particular properties that make MSCs excellent therapeutic agents, can also influence tumor progression. MSCs are able to release multiple agents with pro- and anti-tumor effects, which affect survival, proliferation and angiogenesis among other cell functions. These paracrine agents can be directly secreted into the tumor milieus or secreted via EVs. Furthermore, MSCs can differentiate into CAFs to support tumor progression. Abbreviations: bFGF, basic fibroblast growth factor; BMP, bone morphogenetic protein; CAF, cancer-associated fibroblasts; HGF, hepatocyte growth factor; EVs, extracellular vesicles; IGF1, insulin like growth factor 1; IL6, interleukin 6; IL8, interleukin 8; IL10, interleukin 10; INFγ, interferon gamma; IDO, indoleamine 2,3-dioxygenase; NK, natural killer; PTEN, phosphatidylinositol 3,4,5-trisphosphate 3-phosphatase; PDGF, platelet derived growth factor; TRAIL, TNF-related apoptosis-inducing ligand; TGFβ, transforming growth factor; VEGF, vascular endothelial growth factor.

The therapeutic benefits of MSCs have prompt their use in cell-based strategies to treat different diseases, including cancer. Similar to damaged tissues, tumors exert chemoattractant effects on MSCs that influence their recruitment to tumor sites (Figure 2). The CXCL12/CXCR4 axis is one of the most frequently studied signaling pathways in the mobilization of MSCs to tumor microenvironment (Gao et al., 2009; Xu et al., 2009; Lourenco et al., 2015; Wobus et al., 2015; Kalimuthu et al., 2017). However, the ability of MSCs to migrate toward cancerous tissue is also controlled by other agents, including diffusible cytokines such as IL8, growth factors such as TGFβ1 or platelet derived growth factor (PDGF), and extracellular matrix molecules such as matrix metalloproteinase 2 (MMP-2) (Nakamizo et al., 2005; Birnbaum et al., 2007; Bhoopathi et al., 2011). Once the tumor niche is reached, MSCs interact with cancer cells via direct and indirect mechanisms that affect tumor development (Figure 3). The paracrine function of MSCs is one of the main mechanisms involved in cancer regulation and is mediated by multiple factors, including growth factors and cytokines. These paracrine factors affect cellular processes involving tumor cell cycle (i.e., cell proliferation), cell survival, angiogenesis, and immunosuppression/immunomodulation, allowing MSCs to regulate cancer. The paracrine agents can be directly secreted into the extracellular space or packaged into extracellular vesicles to be spread in the tumor milieus (Rani et al., 2015). The interaction of MSCs with tumor cell cycle is the most commonly accepted process by which MSCs exert their therapeutic effects (Fathi et al., 2019). By inhibiting proliferation-related signaling pathways, such as the phosphatidylinositol 3-kinase/protein kinase B (PI3K/AKT), MSCs can induce cell cycle arrest and reduce cancer growth (Lu et al., 2019). In addition, MSCs can undergo differentiation into other cell types, such as cancer-associated fibroblasts (CAFs), to directly contribute to cancer progression (Jotzu et al., 2011; Barcellos-de-Souza et al., 2016; Aoto et al., 2018) (Figure 3).

Accumulating evidences indicate that the cross-talk between MSCs and tumor cells results in both pro-tumor and anti-tumor effects, raising safety concerns for clinical application in oncology (Barkholt et al., 2013) (Figure 3). The discrepancies in the ability of MSCs to promote or suppress tumor development may be attributable to differences in experimental tumor models, MSC tissue source, dose or timing of the MSC treatment, cell delivery method, control group chosen, and other experimental conditions (Bortolotti et al., 2015; Bajetto et al., 2017). In this regard, a study demonstrated that direct (cell-to-cell contact) or indirect (released soluble factors) interaction between umbilical cord MSCs and glioblastoma stem cells produces divergent effects on cell growth, invasion and migration (Bajetto et al., 2017). Additionally, the application of MSCs for cancer patients is a more complex situation in which other factors have to be taken into consideration. For instance, the pathological conditions of each patient may induce cellular and molecular changes in MSCs that interfere with their therapeutic effects (Capilla-Gonzalez et al., 2018; Perez et al., 2018; Rivera et al., 2019). We must, therefore, be cautious in the conclusions we draw from a single study regarding the therapeutic effects of MSCs in cancer.

### Pro-tumor Activity

The pleiotropic effects of MSCs that promote tissue repair and regeneration may also confer pro-tumor functions to these cells. For instance, metastatic human breast carcinoma cells were found to induce the secretion of the chemokine (C-C motif) ligand 5 (CCL5) from MSCs, which enhanced tumor invasion (Karnoub et al., 2007). Seminal reports demonstrated that MSCs are also able to inhibit apoptosis in tumor cells by secreting pro-survival factors such as VEGF and basic fibroblast growth factor (bFGF) (Konig et al., 1997; Dias et al., 2002).

Numerous studies converged on the finding that MSCs contribute to cancer pathogenesis by releasing inflammatory factors that promote immunosuppressive effects. For example, an *in vitro* study showed that MSCs isolated from gastric tumors mediate cancer progression through secretion of Interleukin 8 (IL8) (Li et al., 2015), a pro-inflammatory chemokine that favors the recruitment of leukocytes. It is known that recruited leukocytes, such as macrophages and neutrophils, facilitate cancer initiation and progression (Guo et al., 2017; Powell et al., 2018). Similarly, MSCs are able to secrete TGFβ that promotes macrophages infiltration at the tumor site and facilitates tumor escape from immune surveillance (Kim et al., 2006; Byrne et al., 2008).

Compelling evidences indicate that MSCs can also support tumor angiogenesis, an essential process in cancer progression that supplies tumors with oxygen and nutrients. For instance, MSCs recruited in breast and prostate tumors were found to increase the expression of angiogenic factors, including TGFβ, VEGF and Interleukin 6, which contribute to tumor growth and vascularization (Zhang et al., 2013). Similarly, a correlation between increased expression of TGFβ1 and higher microvessel density was observed in hepatocellular carcinomas of mice receiving intravenous injections of human MSCs (Li et al., 2016). This study further supported that MSCs may enhance tumor angiogenesis via TGFβ.

Furthermore, MSCs can also respond to soluble factors secreted from cancer cells and differentiate into CAFs, a cell type within the tumor microenvironment capable of promoting tumorigenesis (Mishra et al., 2008). In particular, TGFβ secreted from cancer cells plays a key role in the differentiation of MSCs into CAFs (Jotzu et al., 2011; Barcellos-de-Souza et al., 2016; Aoto et al., 2018). It is known that the transition of MSCs into CAFs contributes to tumor progression in part by their active secretome, which includes immune-modulating agents (CXCL12, Granulocyte Macrophage Colony-Stimulating Factor), pro-angiogenic factors (VEGF, TGFβ, PDGF), pro-survival factors (Hepatocyte Growth Factor, Insulin like Growth Factor 1, Interleukin 6), and extracellular matrix modulators (MMP, Tissue Inhibitor of Metalloproteinases) among others (Kalluri, 2016). Cell engulfment has also been identified as an interaction process between MSCs and cancer cells that enhances tumor aggressiveness. A recent report demonstrated that breast cancer cell engulfment of MSCs leads to changes in the transcriptome profile of tumor cells, mainly associated with oncogenic pathways (Chen et al., 2019). This MSC engulfment enhances epithelial-to-mesenchymal transition, stemness, invasion, and metastasis of breast cancer (Chen et al., 2019).

### Anti-tumor Activity

Although compelling evidences show a pro-tumorigenic role of MSCs, these cells also have potent tumor suppressive effects that have been exploited as cancer therapeutics. Previous studies have demonstrated that MSCs release cytotoxic agents, such as TNF-Related Apoptosis-Inducing Ligand (TRAIL) that selectively induces apoptosis in different types of cancer (Wiley et al., 1995; Hao et al., 2001; Takeda et al., 2001; Akimoto et al., 2013). Recently, a report indicated that bone marrow MSCs promote apoptosis and suppress growth of glioma U251 cells through downregulation of the PI3K/AKT signaling pathway (Lu et al., 2019). Similarly, intravenously transplanted MSCs were found to suppress tumor growth by blocking AKT activation in a Kaposi sarcoma mouse model (Khakoo et al., 2006). In mammary carcinomas, umbilical cord MSCs attenuated cell growth and triggered apoptosis through inhibiting ERK1/2 and AKT activation (Ganta et al., 2009). The Wnt signaling pathway has also been involved in the ability of MSCs to inhibit tumor cell proliferation (Qiao et al., 2008a, b). A mechanistic study of the inhibitory effect of MSCs on breast cancer cells demonstrated that the protein Dickkopf-1 (Dkk-1) released from MSCs blocks tumor growth via depression of Wnt signaling (Qiao et al., 2008a).

In contrast to investigations describing the pro-angiogenic effect of MSCs (Zhang et al., 2013; Li et al., 2016), the anti-tumor activity of MSCs via inhibition of tumor angiogenesis has also been documented. A study reported that bone marrow MSCs restrict vascular growth in ΔGli36 glioma xenograft through downregulation of the PDGF/PDGFR axis (Ho et al., 2013). In particular, the expression of PDGF-BB protein was significantly reduced in tumor lysates when treated with MSCs, which correlated with reduced levels of activated PDGFR-β and the active isoform of its downstream target AKT (Ho et al., 2013). In a melanoma mouse model, transplanted MSCs inhibited angiogenesis in a concentration-dependent manner, leading to a reduced tumor growth (Otsu et al., 2009). Confirmatory *in vitro* studies suggested that the anti-angiogenic effect was due to MSC-induced capillary degeneration (Otsu et al., 2009).

Furthermore, MSCs have elicited anti-tumor immune responses through released inflammatory mediators, such as the multifunctional cytokine TGFβ. Similar to several signaling molecules, TGFβ plays a dual role in cancer development (Bierie and Moses, 2006). Besides the aforementioned pro-tumor functions, TGFβ signaling exhibits suppressive effects in cancer (Dong et al., 2007; Guasch et al., 2007). In fact, while the expression of the type III TGFβ receptor (TβRIII) decreases during breast cancer progression, restoring TβRIII expression suppresses tumorigenicity (Dong et al., 2007).

## MSCs as Carriers of Anti-Cancer Payloads

Over the past decade, research efforts have focused on investigating the potential of stem cells as Trojan horses to selectively deliver anti-cancer payloads to tumor cells. In this context, MSCs have attracted much attention as therapeutic carriers due to their inherent capacity to migrate to tumor sites. Genetic engineering is one of the most common strategies used to produce MSCs delivering tumor-suppressing agents into cancer cells. Typically, MSCs have been genetically modified with viral particles to express cytokines, such as Interferon β (INFβ) (Studeny et al., 2002; Shen et al., 2016). It has been reported that human umbilical cord MSCs transduced with adenoviral vectors expressing IFNβ effectively inhibit the growth of breast cancer cells through induction of apoptosis (Shen et al., 2016). Interleukins are another group of cytokines used as tumor-suppressing agents (Chen et al., 2008; Liu et al., 2018). A recent study using lentiviral transductions showed that human umbilical cord MSCs expressing interleukin-18 inhibit the proliferation and metastasis of breast cancer in mice (Liu et al., 2018). Genetically engineered MSCs with TRAIL have also shown strong anti-tumor activity in different types of cancer (Ciavarella et al., 2012; Fakiruddin et al., 2014; Guo et al., 2016; Jiang et al., 2016). In a fascinating study, X. Jiang and colleagues developed a non-viral method using nanoparticles to produce human MSCs engineered to express the suicide protein TRAIL for targeting and eradicating intracranial gliomas in mice (Jiang et al., 2016). When transplanted in a mouse model of orthotopic patient-derived glioblastoma xenografts, TRAIL-expressing MSCs inhibited tumor growth, induced apoptosis, reduced the occurrence of microsatellites, and extended animal survival.

Beside cytokines, several other proteins have been used as tumor-suppressing agents in MSC engineering for cancer therapy. For instance, Bone Morphogenetic Protein 4 (BMP4)-expressing MSCs were found to efficiently suppress tumor growth and prolong survival of glioma-bearing mice (Li et al., 2014; Mangraviti et al., 2016). Similarly, MSCs modified to express the tumor-suppressor gene Phosphatidylinositol 3,4,5-Trisphosphate 3-Phosphatase (PTEN) induced cytotoxicity of glioma cells (Guo et al., 2016).

MicroRNAs (miRs) have gained special interest in cancer therapy because of their ability to modulate post-transcriptional gene expression. It is known that MSCs express a variety of miRs that can be packaged into extracellular vesicles, and delivered to neighboring cells to exert therapeutic effects (Collino et al., 2014). Taking advantage of this property, MSCs have been engineered to carry specific miRs with anti-cancer properties (Lee et al., 2013; Lang et al., 2018; Sharif et al., 2018; Li X. et al., 2019). For instance, lentiviral vectors were used to engineer MSCs to produce extracellular vesicles carrying high levels of miR-124a, which had an effective anti-tumor action in multiple patient-derived glioma stem cell lines (Lang et al., 2018).

The loading of MSCs with oncolytic viruses has being used as an effective anti-tumor therapy. MSCs infected with the oncolytic adenovirus ICOVIR5 provided therapeutic benefit for the treatment of lung carcinoma in mice, through inhibition of tumor growth and promotion of T cell recruitment to the tumors (Rincon et al., 2017). Similarly, MSCs carrying the oncolytic adenovirus CRAd5/F11 inhibited tumor progression in a subcutaneous murine xenograft model of colorectal cancer (Guo et al., 2019). Different variants of the oncolytic herpes simplex virus has also been used to arm MSCs that effectively track metastatic tumor lesions and prolong survival of mice with brain metastatic melanomas (Du et al., 2017).

Another cellular Trojan horse that has been used for cancer treatment is MSCs loaded with anti-cancer drugs. For instance, an *in vitro* study determined that the conditioned media from gingival papilla-derived MSCs primed with Paclitaxel, Doxorubicin or Gemcitabine inhibit squamous carcinoma growth (Cocce et al., 2017). Paclitaxel-loaded MSCs also exhibited anti-tumor effects in glioma-bearing rats (Pacioni et al., 2015). Latest investigations have focused on the development of strategies to improve the payload and delivery capacity of MSCs. In this sense, nanoparticles are a promising approach to increase the anti-tumor efficacy of MSCs loaded with anti-cancer drugs (Layek et al., 2018; Wang et al., 2018; Moku et al., 2019). Drug-encapsulated nanoparticles offer multiple therapeutic benefits by providing preferential accumulation at the target site, preventing burst release and reducing side effects.

## Limitations of Msc-Based Therapies for Cancer: a Challenge for Biomaterials

The use of engineered MSCs has emerged as a new therapeutic paradigm to treat cancer. However, efficient engraftment and survival of delivered MSCs remains a potential obstacle that limits their therapeutic application. Biomaterials are used in cell therapy as scaffolds that improve the retention of transplanted cells in specific sites to treat different pathologies. This combined use of biomaterials and stem cells allows from the treatment of restricted defects to the repair and replacement of entire organs (Zeng et al., 2015; Wang et al., 2017; Diomede et al., 2018). In the cancer research area, a recent study described a method for the delivery of therapeutic MSCs on biomaterials to treat postoperative brain cancer (Sheets et al., 2018). This approach bases on the implantation of biodegradable fibrin scaffolds seeded with MSCs into the resection cavity to eradicate residual tumor cells in patients receiving surgical removal, with the ultimate goal of increasing cancer-free survival. Another study used cryogel-housed MSCs that were engineered to release anti-CD33-anti-CD3 bispecific antibody for effective immunotherapy in acute myeloid leukemia (Aliperta et al., 2017). In addition to scaffolds, biomaterials are used to encapsulate cells, protecting them from the host while allowing the diffusion of nutrients and therapeutic agents. Microcapsules designed with alginate, cellulose and agarose have shown benefits in cell-based anti-cancer therapies (Pelegrin et al., 1998; Sakai et al., 2005; Schwenter et al., 2011; Johansson et al., 2013). The group of Simone P. Niclou demonstrated that the interstitial delivery of alginate-encapsulated cells expressing the soluble form of the leucine-rich repeats and immunoglobulin-like domains 1 (Lrig1) inhibited tumor growth in orthotopic patient-derived glioblastoma xenografts mouse model (Johansson et al., 2013).

Biomaterials can be designed to have their own anti-cancer activities. Among biomaterials used in oncology, Gliadel represents one of the major success in the development of interstitial therapies for brain cancer (Brem and Gabikian, 2001). Gliadel is a biodegradable medicinal implant made of polifeprosan that is inserted into the resection cavity and slowly releases the anti-cancer agent carmustine over 2–3 weeks. The use of Gliadel in patients receiving surgical removal of brain tumors is associated with moderated survival benefits (Bregy et al., 2013). Latest investigations have discovered a thermo-responsive biodegradable paste that allows delivering of multiple anti-cancer agents with improved results in glioma patients survival (Smith et al., 2019). Once optimized the composition and design of biomaterials, it may be possible to use them in combination with stem cells to release anti-cancer agents in a cooperative manner. Therefore, the application of biomaterial in MSC-based therapies is a potential approach for the treatment of cancer that merits further investigation.

## Clinical Application of MSCs for Cancer Therapy

The last decade has witnessed a rapid development of cell-based therapies for oncological application, being MSCs at the forefront of this new tendency. Aside from their anti-cancer effects, MSCs are of special relevance for personalized cell-based therapies because they can be easily obtained with minimally invasive procedures and rapidly large-scale expanded (Escacena et al., 2015). To date, 25 clinical trials are registered on ClinicalTrials.gov aimed to use MSCs in various cancer conditions. Among these studies, 14 trials are using MSCs as therapeutic agent to directly treat cancer (Table 1). Most of these trials are ongoing phase 1 or 2 studies that are evaluating the safety and efficacy of MSC application in cancer patients. Of special note is a completed phase I/II clinical trial from 2013 that investigated the use of bone marrow-derived autologous MSCs infected with the oncolytic adenovirus ICOVIR5 (CELYVIR) to treat metastatic and refractory solid tumors in children and adults (NCT01844661). This exploratory study evaluated the adverse effects after intravenous infusions of CELYVIR (time frame: 48 h after each infusion) and the clinical outcome (time frame: up to 2 months after the last infusion). The authors concluded that multidoses of CELYVIR have an excellent safety profile and beneficial anti-tumor effects (Garcia-Castro et al., 2010; Melen et al., 2016). Interestingly, they documented a complete remission in one pediatric case 3 years after CELYVIR treatment (Garcia-Castro et al., 2010).

**TABLE 1:** Clinical studies using MSC-based therapies for cancer treatment.

**NCT Number**	**Purpose**	**Condition**	**Therapeutic agent**	**Phase**	**Start date**	**Status**	**Locations**
NCT03896568	To determine the maximal tolerated and toxicity of allogeneic bone marrow-derived MSCs loaded with the oncolytic adenovirus DNX-2401 (BM-MSCs-DNX2401)	Glioma	BM-MSCs-DNX2401	I	2019	Recruiting	United States
NCT03608631	To determine the maximal tolerated and toxicity of MSC-derived exosomes loaded with KrasG12D siRNA (iExosomes)	Pancreatic cancer	iExosomes	I	2019	Not yet recruiting	United States
NCT03298763	To evaluate the safety and anti-tumor activity of MSCs genetically modified to express TRAIL (MSC-TRAIL)	Adenocarcinoma of lung	MSC-TRAIL	I, II	2019	Recruiting	United Kingdom
NCT03184935	To determine the safety and efficacy of human umbilical cord-derived MSCs (UC-MSC)	Myelodysplastic syndromes	UC-MSC	I, II	2017	Unknown	China
NCT02530047	To find the highest tolerable dose of bone marrow-derived MSCs expressing INFb (BM-MSC-INFβ) that can be given To patients with ovarian cancer and to test their safety	Ovarian cancer	BM-MSC-INFβ	I	2016	Active, not recruiting	United States
NCT02181478	To evaluate feasibility and safety of combining intra-osseous umbilical cord blood hematopoietic stem cells (UC-HSC) and MSC	Hematologic malignancies	MSCs UC-HSC	I	2015	Recruiting	United States
NCT02068794	To study the side effects and best dose of adipose tissue-derived MSCs infected with oncolytic measles virus encoding thyroidal sodium iodide symporter (AdMSC-MV-NIS)	Ovarian cancer	AdMSC-MV-NIS	I, II	2014	Recruiting	United States
NCT02079324	To determine maximum tolerable dose, safety and efficacy of intratumoral injected GX-051	Head and neck cancer	GX-051	I	2014	Unknown	Korea
NCT02270307	To evaluate the effectiveness of the use of MSCs and cyclophosphamide	Hematological malignancies	MSCs and cyclophosphamide	II, III	2014	Unknown	Russian Federation
NCT01983709	To evaluate home of bone marrow-derived MSCs (BM-MSCs) to sites of prostate cancer after systemic administration	Prostate cancer	BM-MSCs	I	2013	Terminated	United States
NCT01844661	To evaluate the safety of bone marrow-derived autologous MSCs infected with ICOVIR5 (CELYVIR) in children and adults with metastatic and refractory solid tumors	Solid tumors metastases	CELYVIR	I, II	2013	Completed	Spain
NCT01129739	To evaluate the safety and efficacy of MSCs derived from human umbilical cord/placenta (UC/PL-MSC) at a dose of 1.0E + 6 MSC/kg	Myelodysplastic syndromes	UC/PL-MSC	II	2010	Unknown	China
NCT01092026	To determine the feasibility of umbilical cord blood hematopoietic stem cell (UCB-HSC) transplantation with co-infusion of third party MSCs	Hematological malignancies	UCB-HSC with MSCs	I, II	2010	Unknown	Belgium
NCT01045382	To evaluate the capacity of MSCs to improve 1-year overall survival of patients transplanted with HLA-mismatched allogeneic hematopoietic cells	Hematological malignancies	MSCs	II	2010	Recruiting	Belgium

Furthermore, there are nine registered clinical trials that evaluate the application of MSCs to treat a variety of side effects of cancer treatments, such as cardiomyopathy due to anthracyclines (NCT02509156), xerostomia due to radiotherapy (NCT03874572), cisplatin-induced acute renal failure (NCT01275612), erectile dysfunction after prostatectomy (NCT01089387) or radiation-induced hemorrhagic cystitis (NCT02814864). Therefore, MSC-based therapies stand as a good therapeutic option not only to directly target cancer, but also to minimize the side effects of cancer treatments. Consequently, there are two types of participants that would be potential candidates in a clinical trial of MSC-based therapy for cancer; (1) patients with cancer that are receiving or not receiving treatment, and (2) cancer survivors that experience side effects of oncological treatments. Inclusion criteria for these individuals should be either to be diagnosed with cancer, to suffer side effects of cancer treatments or both. Despite advances are being achieved, the lack of published results involving clinical studies hinders the development of further advances in the therapeutic application of MSCs.

## Concluding Remarks and Future Perspectives

Mesenchymal stem cells are widely used in the treatment of various diseases due to their ability to home to damaged tissues, their ability to differentiate into various cell types and their pleiotropic effects. However, the therapeutic use of MSCs for cancer has been hampered by contradictory results describing both anti- and pro-tumor effects in preclinical studies. Despite this reality, latest MSC-based therapies bring new hope to cancer patients by offering highly effective anti-cancer treatments in a personalized manner. Among MSC-based therapies, the use of MSCs as Trojan horses to deliver therapeutic factors represents an important step forward to a more efficient cancer treatment. The next challenge is to better understand the interaction between MSCs and cancer cells to improve the clinical safety of MSC-based therapeutic approaches. In this context, the use of MSC-derived extracellular vesicles as a cell-free therapy has emerged as a promising option that circumvent the safety concerns associated with the use of live cells. Further research will shed light on the challenges facing cell-free therapy for cancer. We are definitely moving closer to generate a safe and effective medicinal product for cancer that will improve survival and quality of life of patients suffering this devastating disease.

## Author Contributions

VC-G conceived the manuscript. All authors contributed to the manuscript revision and approved the submitted version.

## Conflict of Interest

The authors declare that the research was conducted in the absence of any commercial or financial relationships that could be construed as a potential conflict of interest.
